# Left Ventricular SGLT1 Protein Expression Correlates with the Extent of Myocardial Nitro-Oxidative Stress in Rats with Pressure and Volume Overload-Induced Heart Failure

**DOI:** 10.3390/antiox10081190

**Published:** 2021-07-26

**Authors:** Alex Ali Sayour, Mihály Ruppert, Attila Oláh, Kálmán Benke, Bálint András Barta, Eszter Zsáry, Haoran Ke, Eszter Mária Horváth, Béla Merkely, Tamás Radovits

**Affiliations:** 1 Heart and Vascular Center, Department of Cardiology, Semmelweis University, Városmajor Str. 68, H-1122 Budapest, Hungary; ruppert.mihaly@med.semmelweis-univ.hu (M.R.); olah.attila@med.semmelweis-univ.hu (A.O.); benke.kalman@med.semmelweis-univ.hu (K.B.); barta.balint_andras@med.semmelweis-univ.hu (B.A.B.); eszterzsary@gmail.com (E.Z.); merkely.bela@med.semmelweis-univ.hu (B.M.); radovits.tamas@med.semmelweis-univ.hu (T.R.); 2 Department of Physiology, Semmelweis University, Tűzoltó Str. 37-47, H-1094 Budapest, Hungary; simonke0430@gmail.com (H.K.); horvath.eszter@med.semmelweis-univ.hu (E.M.H.)

**Keywords:** sodium-glucose cotransporter 2 inhibitor, sodium-glucose cotransporter 1, myocardial nitro-oxidative stress, NADPH oxidase 4, heart failure, pressure overload, volume overload

## Abstract

Myocardial sodium-glucose cotransporter 1 (SGLT1) has been shown to be upregulated in humans with heart failure (HF) with or without diabetes. In vitro studies have linked SGLT1 to increased nitro-oxidative stress in cardiomyocytes. We aimed to assess the relation between left ventricular (LV) SGLT1 expression and the extent of nitro-oxidative stress in two non-diabetic rat models of chronic heart failure (HF) evoked by either pressure (TAC, *n* = 12) or volume overload (ACF, *n* = 12). Sham-operated animals (Sham-T and Sham-A, both *n* = 12) served as controls. Both TAC and ACF induced characteristic LV structural and functional remodeling. Western blotting revealed that LV SGLT1 protein expression was significantly upregulated in both HF models (both *p* < 0.01), whereas the phosphorylation of ERK1/2 was decreased only in ACF; AMPKα activity was significantly reduced in both models. The protein expression of the Nox4 NADPH oxidase isoform was increased in both TAC and ACF compared with respective controls (both *p* < 0.01), showing a strong positive correlation with SGLT1 expression (*r* = 0.855, *p* < 0.001; and *r* = 0.798, *p* = 0.001, respectively). Furthermore, SGLT1 protein expression positively correlated with the extent of myocardial nitro-oxidative stress in failing hearts assessed by 3-nitrotyrosin (*r* = 0.818, *p* = 0.006) and 4-hydroxy-2-nonenal (*r* = 0.733, *p* = 0.020) immunostaining. Therefore, LV SGLT1 protein expression was upregulated irrespective of the nature of chronic hemodynamic overload, and correlated significantly with the expression of Nox4 and with the level of myocardial nitro-oxidative stress, suggesting a pathophysiological role of SGLT1 in HF.

## 1. Introduction

Sodium-glucose cotransporter 1 (SGLT1) has recently been identified as a major glucose transporter in the heart [1]. We previously showed that left ventricular (LV) SGLT1 is upregulated in patients with end-stage heart failure (HF) compared with non-failing controls [2]. In these patients, SGLT1 expression showed a significant correlation with the extent of LV dilation and systolic dysfunction independent of age, sex, and body mass index [2]. Knockout or knockdown of SGLT1 prevents LV pathological remodeling in murine models of pressure overload [3] or type 2 diabetes mellitus (T2DM) [4,5], whereas humans with functionally limited SGLT1 have substantially lower risk of developing HF in the long-term [6]. These suggest that SGLT1 is causally implicated in the development of HF.

The mechanisms responsible for upregulation of SGLT1 in chronic HF are unclear, as are the actions through which SGLT1 contributes to myocardial damage. Extracellular signal-regulated protein kinase (ERK) 1/2 and adenosine monophosphate-activated protein kinase (AMPK) have been implicated as positive regulators of SGLT1 expression in the heart during myocardial ischemia-reperfusion injury [7], whereas genetic overactivation of myocardial AMPK and resultant cardiomyopathy is associated with upregulation of SGLT1 [8]. Previous studies showed that SGLT1 activates nicotinamide adenine dinucleotide phosphate (NADPH) oxidase in cell cultures of cardiomyocytes, increasing the level of nitro-oxidative stress [7,9].

In the present study, we hypothesized that myocardial LV SGLT1 protein expression is upregulated in non-diabetic rats with pressure and volume overload-induced chronic HF and correlates with the extent of myocardial nitro-oxidative stress.

## 2. Materials and Methods

### 2.1. Experimental Animals

A total of 48 male Wistar rats (purchased from Toxi-Coop; Budapest, Hungary) were kept under standard conditions (22 ± 2 °C with 12 h light/dark cycles) and were allowed access to laboratory rat diet and water ad libitum during the experimental period. Prior to experimentations, rats were allowed to acclimatize for one week. The present investigation conformed to the EU Directive 2010/63/EU and to the Guide for the Care and Use of Laboratory Animals published by the US National Institutes of Health (NIH Publication No. 85-23, revised 1996). The study was approved by the Scientific Ethical Committee on Animal Experimentation (Hungary) and by the Institutional Ethics Committee of Semmelweis University (Reference No. PEI/001/2374-4/2015).

#### 2.1.1. Model of Pressure Overload-Induced Heart Failure

Three-week-old (50–100 g) male Wistar rats underwent transverse aortic constriction (TAC) to induce chronic progressive pressure overload for 14 weeks resulting in HF, as described earlier [10,11]. In brief, anesthesia was induced by placing the animals in a chamber filled with 5% isoflurane. Then, animals were intubated, and anesthesia was maintained with a small animal respirator using 2% isoflurane (mixed in pure oxygen). Core temperature (37 ± 0.5 °C) was kept constant by placing the rats in a supine position on a controlled heating pad. Left anterolateral thoracotomy was performed in the second intercostal space; next, the aortic arch was isolated and constricted to match the size of the external diameter of a 21-gauge needle between the innominate artery and the left common carotid artery. Finally, the thorax was closed and the skin layers were sutured. The wound was carefully disinfected, tramadol (10 mg/kg) and physiological saline were subcutaneously injected shortly after weaning the animals off the respirator.

Age and sex-matched control animals (Sham-T) underwent the same procedure as above, except the aortic arch was not constricted (i.e., no pressure overload).

#### 2.1.2. Model of Volume Overload-Induced Heart Failure

Six-week-old (150–200 g) male Wistar rats underwent shunting of the abdominal aorta and the inferior vena cava to induce chronic progressive volume overload for 24 weeks resulting in HF, as described earlier [10,11]. Briefly, anesthesia was induced by placing the animals in a chamber filled with 5% isoflurane. Then, anesthesia was maintained by inhalation of 2% isoflurane (mixed in pure oxygen) from an insulated facemask. Core temperature (37 ± 0.5 °C) was kept constant by placing the rats in a supine position on a controlled heating pad. Following thorough shaving of the abdomen and application of strict aseptic measures, a midline laparotomy was performed. After exposing the abdominal aorta and the inferior vena cava, both were clipped transiently distal to the origin of the left renal artery and proximal to the aortic bifurcation. Then, in this isolated section, the anterior aortic wall was punctured (18-gauge needle), followed by advancement through the adjacent venous wall, creating an aortocaval fistula (ACF). Following the establishment of the shunt, the needle was withdrawn and the puncture on the surface of the aorta was sealed using a drop of cyanoacrylate glue. When the ACF was secured, the intestines were replaced, the abdominal muscle layers were sutured, followed by closure of the skin incision. Then, the wound was carefully disinfected, tramadol (10 mg/kg) and physiological saline were subcutaneously injected shortly after suspending the anesthesia.

Age and sex-matched control animals (Sham-A) underwent the same procedure as above, except ACF was not created (i.e., no volume overload).

#### 2.1.3. Experimental Groups

Based on the above, our study comprised four experimental groups:

- Sham-T (*n* = 12): rats undergoing sham operation as controls of TAC and followed for 14 weeks;
- TAC (*n* = 12): rats undergoing TAC and followed for 14 weeks;
- Sham-A (*n* = 12): rats undergoing sham operation as controls of ACF and followed for 24 weeks;
- ACF (*n* = 12): rats undergoing ACF operation and followed for 24 weeks.

### 2.2. Echocardiographic Measurements

The Vivid I (GE Healthcare, Waukesha, WI, USA) echocardiographic imaging system equipped with the GE 12L-RS linear transducer (13 MHz) was used to non-invasively assess the temporal alterations in LV structure and function [10,11]. Prior to measurements, rats were anesthetized in a chamber with 5% isoflurane, then anesthesia was maintained by inhalation of 2% isoflurane (mixed in pure oxygen) from an insulated facemask, while placing the rats in a supine position on a controlled heating pad (maintaining core temperature at 37 ± 0.5 °C throughout the measurements). In order to optimize the acoustic window, the thorax was thoroughly shaved. Images were captured in two-dimensional parasternal long-axis and short-axis views at the mid-papillary level by M.R. The digital images were analyzed offline by a blinded investigator (B.A.B.) using EchoPac (GE Healthcare). The following parameters were obtained from the average of three consecutive cardiac cycles (devoid of breathing movements): LV end-diastolic diameter (LVEDD), LV end-systolic diameter (LVESD), anterior wall thicknesses (AWT) and posterior wall thicknesses (PWT) in diastole (d) and systole (s). Then, LV mass was quantified using the Devereux formula [12], whereas LV end-diastolic volume (LVEDV), LV end-systolic volume (LVESV), and ejection fraction (EF) were calculated according to the Teichholz equation [13].

### 2.3. Left Ventricular Pressure-Volume Analysis

Left ventricular pressure-volume (PV) analysis was performed as previously described [14,15], with slight amendments. In brief, rats were anesthetized in a chamber filled with 5% isoflurane, then following tracheotomy and intubation, anesthesia was maintained by artificial ventilation of 1.5% isoflurane (mixed in pure oxygen). For fluid administration, the left external jugular vein was cannulated. Thereafter, rocuronium bromide (2 mg/kg BW) was administered intraperitoneally to achieve generalized muscle relaxation. A 2F microtip pressure-conductance catheter (SPR-838; Millar Instruments, Houston, TX, USA) was advanced into the ascending aorta through the right common carotid artery. Following stabilization, the catheter was guided into the LV under pressure control. The following parameters were obtained using a PV analysis software (PVAN; Millar Instruments): heart rate, LV end-systolic pressure (LVESP), and time constant of LV pressure decay (Tau). Then, the slope of the end-systolic PV relationship (ESPVR)—a relatively load independent contractility index—was calculated from PV loops registered while transiently reducing preload (achieved by transient occlusion of the inferior vena cava). For analysis, all PV loops were acquired with the ventilator turned off for 5 s and the animal apnoeic (due to generalized muscle relaxation). Then, volume calibration was performed by calculating parallel conductance.

Animals were euthanized at the end of the PV protocol, followed by cannulation of the abdominal aorta. After collection of arterial blood, cold (4 °C) 50 mL Ringer solution was infused retrogradely. After the washout, hearts were excised and weighed, midpapillary cross-sections were obtained and stored in 4% buffered paraformaldehyde (for immunohistochemical analyses). Other parts of the LV were instantly snap-frozen in liquid nitrogen and stored at −80 °C (for molecular measurements). Tibial length was measured post-mortem.

### 2.4. RNA Isolation and Polymerase Chain Reaction

Deep-frozen (~25 mg) myocardial LV tissue samples were homogenized in Buffer RLT (Qiagen, Venlo, The Netherlands) by a tissue homogenizer with constant cooling. Thereafter, total RNA was isolated as per the manufacturer’s protocol using the RNeasy Fibrous Tissue Kit (Qiagen). We quantified RNA concentration photometrically at 260 nm, whereas sample purity was ensured by obtaining 260/280 nm and 260/230 nm optical density ratios of ~2.0, respectively.

The QuantiTect Reverse Transcription Kit (Qiagen) was used as per the manufacturer’s protocol to reverse transcribe RNA into cDNA using 1 μg RNA of each sample. We performed qRT-PCR on StepOnePlus RT PCR System (Applied Biosystems, Foster City, CA, USA) with triplicates of each sample in a volume of 10 μL in each well comprising 1 μL cDNA and 9 μL TaqMan Universal PCR MasterMix plus Taqman Gene Expression Assay (Thermo Fisher Scientific, Waltham, MA, USA) for the following targets: pathological hypertrophy marker β/α-myosin heavy chain (β/α-MHC; β-MHC assay ID: Rn00568328_m1; α-MHC assay ID: Rn00568304_m1), and pro-fibrotic markers, including transforming growth factor-β (TGF-β; assay ID: Rn00572010_m1), connective tissue growth factor (CTGF; assay ID: Rn01537279_g1), and collagen type I alpha 1 (Col1a1; assay ID: Rn01463848_m1).

Within each sample, gene expression data were normalized to that of glyceraldehyde 3-phosphate dehydrogenase (GAPDH; assay ID: Rn01775763_g1), and expression levels were calculated using the C_T_ comparative method. All values were normalized to a positive calibrator (pool of cDNA from the Sham group) expressed as 2^−ΔΔCT^.

### 2.5. Protein Isolation and Western Blotting

Deep-frozen (~25 mg) myocardial LV tissues samples were homogenized in RIPA buffer (Bio-Rad Laboratories, Hercules, CA, USA) containing protease-phosphatase inhibitor cocktail (Roche, Basel, Switzerland) using a tissue homogenizer with constant cooling. The protein concentration of the obtained homogenates was quantified using the BCA assay (Thermo Fisher Scientific). Then, the sample buffer was added to the homogenates, and they were heated for 5 min at 95 °C.

Each sample (40 μg protein) was loaded on 6–12% acrylamide gels and separated with sodium dodecyl sulphate polyacrylamide gel electrophoresis system (Bio-Rad Laboratories). Under dry conditions, gels were blotted onto PVDF membranes followed by blocking at room temperature with bovine serum albumin (5% BSA in Tris-buffered saline Tween 20). Membranes were then incubated overnight (at 4 °C) separately, with the following antibodies (1:1000 in 2.5% BSA): anti-NADPH oxidase 4 (Nox4; ID: ab133303; Abcam, Cambridge, UK); anti-SGLT1 (ID: #5042; Cell Signaling Technology, Danvers, MA, USA); anti-phosphorylated AMPKα catalytic subunit (P-AMPKα, Thr172; ID: #2535); anti-total-AMPKα (ID: #2532); anti-phosphorylated ERK 1/2 (P-ERK1/2, Thr202/Tyr204; ID: #9101); anti-total-ERK1/2 (ID: #9102); anti-phosphorylated acetyl coenzyme-A carboxylase (P-ACC, Ser79; ID: #3661). Following the washing procedure, membranes were incubated for 1h (at room temperature) with horseradish peroxidase-conjugated secondary antibody (1:5000 in 2.5% BSA). Super Signal West Pico Plus (Thermo Fisher Scientific) chemiluminescent substrate was used to develop the immunoreactive protein bands. Thereafter, membranes were incubated with a primary antibody against GAPDH (1:5000 in 2.5% BSA, ID: #5174), which we considered as housekeeping. The Bio-Rad Image Lab Software (Bio-Rad Laboratories) was used to analyze the intensity of the developed bands. Within each sample, the intensity of the bands of the primary targets was normalized to that of the housekeeping GAPDH on the same blot.

### 2.6. Histology and Immunohistochemistry

LV tissues were fixed in 4% neutral buffered paraformaldehyde solution, embedded in paraffin, and 5 µm thick histological sections were sliced. Following deparaffinization, endogenous peroxidase activity was blocked by 3% H_2_O_2_, and 2.5% normal horse serum (Vector Laboratories, Burlingame, CA, USA) was used to prevent non-specific labeling. After overnight incubation with the rabbit polyclonal anti-3-nitrotyrosin (3-NT, nitrosative stress marker) antibody (1:500, Merck Millipore, Burlington, MA, USA) or the rabbit polyclonal anti-4-hydroxy-2-nonenal (4-HNE, oxidative stress marker) (1:100, Abcam, Cambridge, UK) antibody at 4 °C, secondary labeling was achieved by HRP-linked anti-rabbit polyclonal horse antibodies (Vector Laboratories), which was visualized by brown-colored diamino-benzidine (DAB, Vector Laboratories). Images of the immunolabeled LV tissue sections were captured by Nikon Eclipse Ni Microscope (Nikon Instruments, Amstelveen, The Netherlands) with 20× objective lens, using a Nikon DS-RI2 camera (Nikon Instruments) and NIS-Elements BR imaging software (Nikon Instruments). The percentage of positively stained tissue area compared to the total tissue area was measured with ImageJ Software (National Institutes of Health, Bethesda, MA, USA). Following image analysis, blue-colored hematoxylin (Vector Biolabs) was utilized as counterstaining, after which representative images were captured.

Additionally, in separate staining procedures, hematoxylin-eosin staining was performed to visualize cellular structure.

### 2.7. Statistical Analysis

Values are expressed as mean ± standard error of the mean (SEM) for continuous variables. The assumption of normal distribution of the data sets was analyzed using the Shapiro–Wilk test and the predicted probability (P-P) plots. The significance of difference between two groups with normally distributed data was assessed using unpaired Student’s *t*-test with Welch’s correction. When data followed non-normal distribution, the non-parametric Mann–Whitney *U* test was used.

To analyze the temporal development of LV hypertrophy (LV mass), mixed analysis of variance (ANOVA) was conducted without assuming sphericity, including hypothesis testing for the following factors: type of surgery (P_TAC_ or P_ACF_ versus respective Sham); time (P_time_); and their interaction (P_int_). Post hoc analyses at different time-points between the operated and respective sham groups were conducted using Bonferroni correction.

For correlation analysis of two continuous variables, Spearman’s *rho* (*r*_s_) was computed, and 95% confidence intervals were obtained.

GraphPad Prism 8 (GraphPad Software, San Diego, CA, USA) was used to analyze and visualize the data. In all cases, untransformed original datapoints were depicted. For hypothesis testing, a two-tailed *p* < 0.050 value was defined as statistically significant.

## 3. Results

### 3.1. TAC Induced Characteristic LV Structural and Functional Alterations

**Figure 1:**
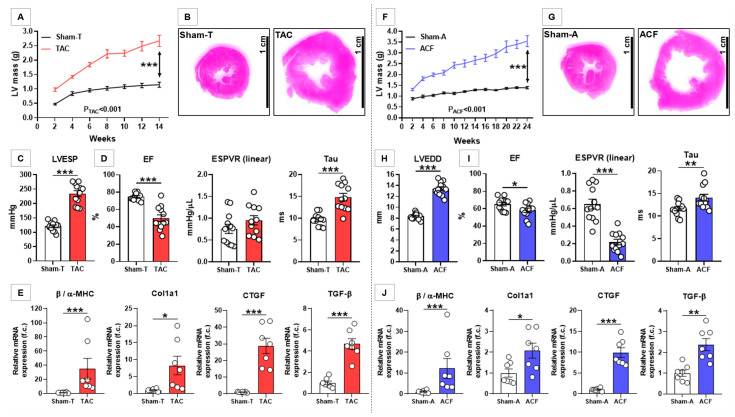
Characterization of pressure and volume overload-induced heart failure. (**A**) Temporal changes in left ventricular (LV) mass according to the Sham-T and pressure-overloaded (TAC) groups. (**B**) Representative histological section at the mid-papillary level of a control (Sham-T) and a TAC heart. (**C**) LV end-systolic pressure (LVESP) in Sham-T and TAC groups. (**D**) LV ejection fraction (EF), end-systolic pressure-volume relationship (ESPVR) and time constant of LV pressure decay (Tau) in Sham-T and TAC groups. (**E**) LV myocardial mRNA expression of pathological hypertrophy markers β/α-myosin heavy chain (β/α-MHC), collagen type I alpha 1 (Col1a1), connective tissue growth factor (CTGF), and transforming growth factor beta (TGF-β). (**F**) Temporal changes in LV mass according to the Sham-A and volume-overloaded (ACF) groups. (**G**) Representative histological section at the mid-papillary level of a control (Sham-A) and an ACF heart. (**H**) LV end-diastolic diameter in Sham-A and ACF groups. (**I**) LV EF, ESPVR and Tau in Sham-A and ACF groups. (**J**) LV myocardial mRNA expression of pathological hypertrophy markers β/α-MHC, Col1a1, CTGF, and TGF-β. In the case of the gene expression data, values are normalized to the mean of the respective Sham group to depict fold changes. In the temporal analysis (LV mass), P_TAC_ and P_ACF_ represents hypothesis testing for the main effect of the type of HF. * *p* < 0.050, ** *p* < 0.010, *** *p* < 0.001.

In animals of the TAC group, chronic pressure overload resulted in severe LV hypertrophy according to gradually increasing LV mass throughout the follow-up period (P_TAC_ < 0.001), which was substantially higher at Week 14 as compared with Sham-T controls (*p* < 0.001) (Figure 1A). The representative section at the mid-papillary level shows marked concentric hypertrophy in TAC (Figure 1B). This was also reinforced by post-mortem organ weight measurements (Table 1), indicating significantly bigger heart weights (HWs) in TAC animals, both in absolute and indexed (to tibial length, TL) terms, respectively (both *p* < 0.001). LV backward failure was evidenced by the significant increment in lung weights (LW) as compared with Sham-T controls (both *p* < 0.001) (Table 1).

At the end of the follow-up, LVESP was significantly higher in those with TAC versus controls (234 ± 11 mmHg vs. 121 ± 5 mmHg, *p* < 0.001) (Figure 1C). There was evidence of moderate LV dilation according to LVEDD values (9.0 ± 0.2 mm vs. 7.6 ± 0.2 mm, *p* < 0.001).

LV systolic function was severely compromised as EF was significantly lower at the end of the experimental period (*p* < 0.001) (Figure 1D). On the contrary, LV contractility (ESPVR) was preserved at the end of the follow-up (Figure 1D), whereas the time-constant of LV pressure decay (Tau) was significantly prolonged in TAC animals, the latter suggesting severe LV diastolic dysfunction (Figure 1D).

The pathological nature of LV hypertrophy in TAC was evidenced by the several fold increase in the LV mRNA expression ratio of β/α-MHC (Figure 1E). Furthermore, the LV mRNA expression of Col1a1 showed a significant upregulation (*p* = 0.040) in line with a several-fold increase in expressions of the profibrotic master regulators CTGF and TGF-β, respectively (both *p* < 0.001) (Figure 1E).

### 3.2. ACF Induced Characteristic LV Structural and Functional Alterations

In the ACF group, chronic volume overload was associated with the development of marked LV hypertrophy, in line with the steady increase in LV mass during the follow-up (P_ACF_ < 0.001), which was significantly increased at Week 24 compared with Sham-A controls (*p* < 0.001) (Figure 1F). The representative section at the mid-papillary level shows marked eccentric hypertrophy in ACF (Figure 1G). The significantly bigger post-mortem absolute and indexed HWs confirmed the development of hypertrophy on the organ level (both *p* < 0.001) (Table 1), whereas the significant increase in LWs demonstrated LV backward failure (both *p* < 0.001) (Table 1).

At the end of the follow-up, LVEDD was significantly higher in ACF rats versus controls (13.4 ± 0.4 mm vs. 8.4 ± 0.2 mm, *p* < 0.001) (Figure 1H) indicating substantial LV dilation.

The significant reduction in EF showed mild systolic dysfunction in ACF rats (*p* = 0.046) (Figure 1I); however, LV contractility was severely compromised according to ESPVR values (*p* < 0.001) (Figure 1I). LV diastolic dysfunction was evidenced by the significant prolongation of Tau (*p* = 0.009) (Figure 1I).

Compared with controls, the several-fold increase in the LV mRNA expression ratio of β/α-MHC (*p* < 0.001), and in the mRNA expressions of Col1a1 (*p* = 0.028), CTGF (*p* < 0.001), and TGF- β (*p* = 0.002), respectively, reinforced the pathological nature of LV hypertrophy in ACF (Figure 1J). Nonetheless, these changes were of much smaller magnitude as compared with TAC hearts.

### 3.3. SGLT1 Protein Expression Was Upregulated Regardless of Type of HF

**Figure 2:**
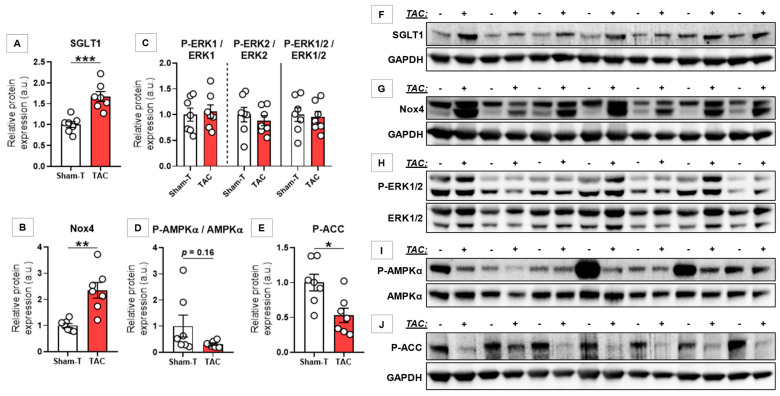
Western blot analysis of left ventricular samples from sham-operated and pressure-overloaded rats with heart failure. (**A–E**) Relative protein expression of left ventricular (LV) sodium-glucose cotransporter 1 (SGLT1); nicotinamide adenine dinucleotide phosphate (NADPH) oxidase isoform Nox4; phosphorylated extracellular matrix-regulated protein kinase 1 and 2 (P-ERK1/2; Thr202/Tyr204), and total ERK1 and 2 ratio separately and in combination; phosphorylated adenosine monophosphate-activated protein kinase α subunit (P-AMPKα; Thr172) and total AMPKα ratio; and phosphorylated acetyl coenzyme-A carboxylase (P-ACC; Ser79). (**F–J**) Cropped full-length blots according to the quantification. Following normalization to corresponding housekeeping (GAPDH), protein expression data were normalized to the mean of the respective Sham group to depict fold changes. * *p* < 0.050, ** *p* < 0.010, *** *p* < 0.001.

In rats with pressure overload-induced HF (TAC), LV SGLT1 protein expression was significantly upregulated (~1.7-fold) as compared with controls (*p* < 0.001) (Figure 2A). Nox4 protein expression showed a similar upregulation (*p* = 0.004) (Figure 2B). As for possible mediators, the activating phosphorylation of separate and combined ERK1/2 was similar between TAC and control animals (Figure 2C), whereas that of AMPKα tended to be lower in TAC (*p* = 0.16) (Figure 2D). However, phosphorylation of ACC at the AMPK-specific Ser79 residue was significantly downregulated in TAC as compared with controls (*p* = 0.011) (Figure 2E). Blots are depicted in Figure 2F–J.

**Figure 3:**
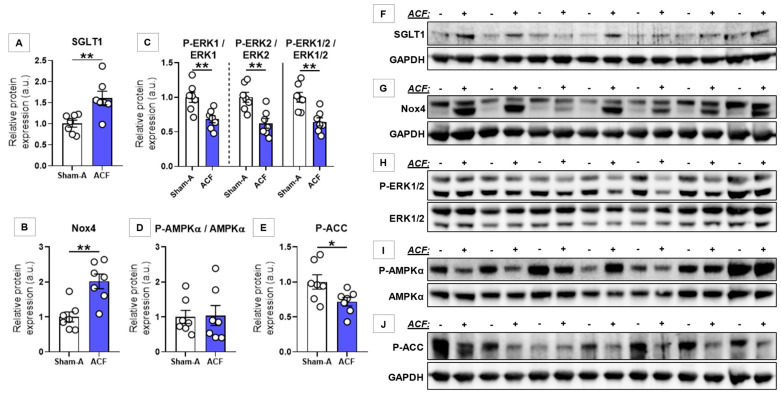
Western blot analysis of left ventricular samples from sham-operated and volume-overloaded rats with heart failure. (**A–E**) Relative protein expression of left ventricular (LV) sodium-glucose cotransporter 1 (SGLT1); nicotinamide adenine dinucleotide phosphate (NADPH) oxidase isoform Nox4; phosphorylated extracellular matrix-regulated protein kinase 1 and 2 (P-ERK1/2; Thr202/Tyr204), and total ERK1 and 2 ratio separately and in combination; phosphorylated adenosine monophosphate-activated protein kinase α subunit (P-AMPKα; Thr172) and total AMPKα ratio; and phosphorylated acetyl coenzyme-A carboxylase (P-ACC; Ser79). (**F–J**) Cropped full-length blots according to the quantification. Following normalization to corresponding housekeeping (GAPDH), protein expression data were normalized to the mean of the respective Sham group to depict fold changes. * *p* < 0.050, ** *p* < 0.010.

Chronic volume overload (ACF) was associated with significant upregulation (~1.6-fold) of LV SGLT1 protein expression compared with controls (*p* = 0.008) (Figure 3A). Similarly, Nox4 was upregulated (*p* = 0.002) (Figure 3B). Unlike in TAC, ACF rats presented with a significantly decreased activating phosphorylation of separate and combined ERK1/2 (*p* = 0.003) (Figure 3C), while that of AMPKα was preserved (Figure 3D). Nonetheless, AMPK-specific phosphorylation of ACC was significantly decreased (*p* = 0.041) (Figure 3E).

### 3.4. SGLT1 Protein Expression Correlates with Nox4 Protein Expression and with the Extent of Myocardial Nitro-Oxidative Stress

**Figure 4:**
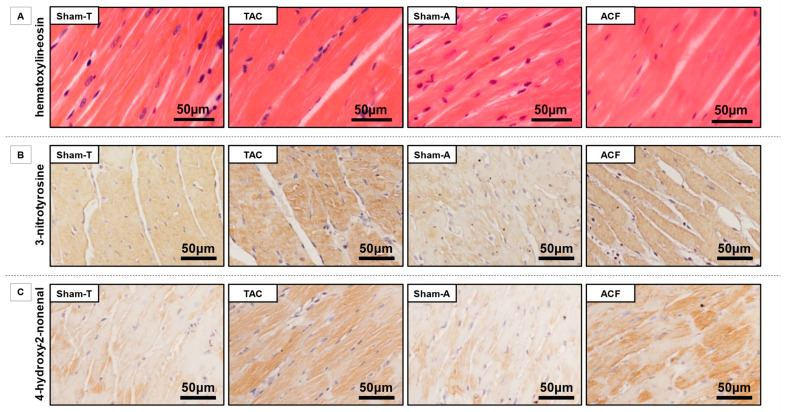
Histological sections from sham-operated and hemodynamically-overloaded hearts. (**A**) Representative LV sections of Sham-T, TAC, Sham-A, and ACF hearts stained with hematoxylin-eosin. (**B**) Representative LV sections of Sham-T, TAC, Sham-A, and ACF hearts with immunohistochemical staining against 3-nitrotyrosine (3-NT), a marker of nitrosative stress. Hematoxylin was used to stain nuclei. Images were captured with 20× objective, scale bars are shown for reference on each representative section. (**C**) Representative LV sections of Sham-T, TAC, Sham-A, and ACF hearts with immunohistochemical staining against 4-hydroxy-2-nonenal (4-HNE), a marker of oxidative stress. Hematoxylin was used to stain nuclei. Images were captured with 20x objective, scale bars are shown for reference on each representative section.

Figure 4A shows the representative hematoxylin-eosin-stained LV sections from Sham-T, TAC, Sham-A, and ACF rat hearts. Immunohistochemical staining against the nitrosative stress marker 3-NT and the oxidative stress marker 4-HNE revealed a higher positivity in the failing hearts compared with respective controls (Figure 4B,C), indicating increased nitro-oxidative stress.

**Figure 5:**
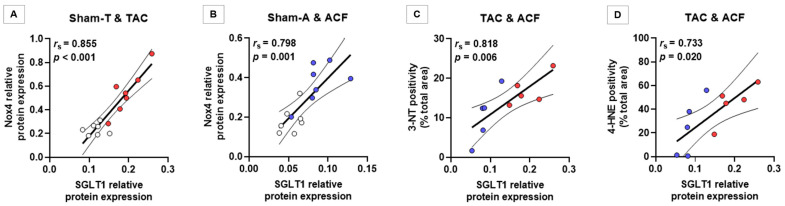
Correlation between left ventricular SGLT1 protein expression, Nox4 protein expression and the extent of myocardial nitro-oxidative stress. (**A**,**B**) Spearman correlation analysis of left ventricular (LV) sodium-glucose cotransporter 1 (SGLT1) protein expression and nicotinamide adenine dinucleotide phosphate (NADPH) oxidase isoform Nox4 protein expression in the Sham-T and pressure-overloaded (TAC), and Sham-A and volume-overloaded (ACF) groups. (**C**) Spearman correlation analysis of western blot-derived LV SGLT1 protein expression and myocardial 3-nitrotyrosine (3-NT) positivity (expressed as percentage of the total area) assessed by immunohistochemical analysis in rats with pressure and volume overload-induced heart failure (TAC and ACF). (**D**) Spearman correlation analysis of western blot-derived LV SGLT1 protein expression and myocardial 4-hydroxy-2-nonenal (4-HNE) positivity (expressed as percentage of the total area) assessed by immunohistochemical analysis in rats with pressure and volume overload-induced heart failure (TAC and ACF).

In the Sham-T and TAC groups, LV SGLT1 protein expression showed a significant, strong correlation with Nox4 protein expression (*r_s_* = 0.855, *p* < 0.001) (Figure 5A). This was also the case with Sham-A and ACF rats (*r_s_* = 0.798, *p* = 0.001) (Figure 5B).

In rats with pressure and volume overload-induced HF, LV SGLT1 protein expression significantly correlated with the extent of myocardial 3-NT positivity (TAC and ACF: *r_s_* = 0.818, *p* = 0.006) (Figure 5C) and with 4-HNE positivity (TAC and ACF: *r_s_* = 0.733, *p* = 0.020) (Figure 5D), indicating a robust association between SGLT1 protein expression and the level of nitro-oxidative stress in HF.

## 4. Discussion

To the best of our knowledge, this is the first study to show that myocardial LV SGLT1 protein expression is significantly upregulated in non-diabetic rats with HF, regardless of whether pressure or volume overload was the predominant underlying pathophysiology. Furthermore, the protein expression of LV SGLT1 strongly correlates with that of the NADPH oxidase isoform Nox4, as well as with the extent of myocardial nitro-oxidative stress.

Although SGLT2 is not expressed in the heart, SGLT1 has recently been identified as a major myocardial glucose transporter alongside the facilitative glucose transporters (GLUT1 and 4) [1,2,16,17,18,19]. Importantly, myocardial SGLT1 has come under scientific interest [20] as SGLT2 inhibitors (with variable selectivity to SGLT2 over SGLT1) [21,22,23,24] and the dual SGLT1/2 inhibitor sotagliflozin [25] were shown to consistently reduce hospitalization for HF in patients with T2DM, independent of antihyperglycemic or diuretic action [26,27,28]. Nonetheless, the pathophysiological role of SGLT1 in cardiac diseases is incompletely understood. Knockout of SGLT1 in non-diabetic mice prevents the development of HF in response to chronic pressure overload (TAC) [3], whereas humans with functionally limiting single nucleotide polymorphisms in the gene encoding SGLT1 are at significantly lower risk of developing HF in the long-term [6]. On the contrary, cardiomyocyte-specific SGLT1 overexpression itself is sufficient to induce severe LV dilation and dysfunction in mice [29]. Therefore, upregulation of SGLT1 might contribute to the development of chronic HF.

Indeed, we previously documented that patients with end-stage HF, due to non-ischemic dilated cardiomyopathy or ischemic heart disease, with or without T2DM exhibited upregulation of LV SGLT1 mRNA and protein expression as compared with non-failing controls [2], in line with other reports [1,18,30]. In these patients, LV SGLT1 expression was independently associated with the extent of LV dilation and systolic dysfunction [2], highlighting the clinical relevance of upregulation of LV SGLT1 in HF. Similarly, previous studies noted that SGLT1 expression was upregulated in non-diabetic small animal models of acute myocardial ischemia-reperfusion injury [7], and permanent left anterior descending coronary artery ligation (model of ischemic heart disease) [1,31], as well as in models of metabolic syndrome/T2DM [1,4,5,30]. Matsushita et al. [3] reported that in non-diabetic mice with pressure overload (TAC) for 6 weeks, LV mRNA expression of SGLT1 was significantly upregulated, together with those of the pathological markers CTGF and Col1a1. Further to this, we demonstrate for the first time that TAC-induced chronic HF for 14 weeks is associated with increased LV SGLT1 expression on the protein level, in line with upregulation of the mRNA expression of pathological hypertrophy markers (β/α-MHC ratio, CTGF, Col1a1, and TGF-β). Moreover, our study is the first to document that chronic volume-overload induced HF is also associated with increased SGLT1 protein expression. Therefore, SGLT1 expression might be upregulated irrespective of whether HF was evoked predominantly by chronic pressure or volume overload, suggesting that this upregulation of SGLT1 might be a shared pathology in HF. Due to the fact that pressure overload (TAC) fails to induce pathological hypertrophy, myocardial fibrosis, LV dilation and dysfunction in mice with global SGLT1 knockout [3], the upregulation of SGLT1 seems to be causally implicated in adverse remodeling in response to chronic hemodynamic overload.

Data are scarce regarding the signal transduction involved in the upregulation of SGLT1 in chronic HF. Recently, ERK1/2 has been suggested as a positive regulator of myocardial SGLT1 expression during acute ischemia-reperfusion injury [7]. In the present study, despite comparable upregulation of myocardial SGLT1 protein expression in pressure and volume overload-induced chronic HF, the activating phosphorylation of ERK1/2 was preserved in TAC hearts (pressure overload for 14 weeks), whereas it was significantly downregulated in those with ACF (volume overload for 24 weeks). As for pressure overload, previous studies showed that ERK1/2 activity is initially increased during the first 6 weeks [3,32,33], declining thereafter to the level of controls [33]. On the contrary, volume-overloaded hearts exhibit preserved ERK1/2 activity initially [32], which gets substantially downregulated at week 2 [34]. There is strong evidence that suppression of myocardial ERK1/2 activity predisposes the LV to dilation (rather than concentric growth) [35], explaining that in the present study mildly dilated TAC hearts had preserved ERK1/2 activity, while those with severely dilated ACF hearts had compromised ERK1/2 activity. Based on these observations, preserved ERK1/2 activity in TAC animals might translate into the loss of the early putative increment in ERK1/2 activity, and could be viewed as diminishing, similarly to ACF hearts. In our previous work, ERK1/2 activity was preserved in those with end-stage HF due to hypertrophic cardiomyopathy without significant LV dilation, however, it was depressed in those with HF and severe LV dilation compared with non-failing controls [2]. Interestingly, in these patients, the increase in SGLT1 expression was proportional to the reduction in the activating phosphorylation of ERK1/2 [2]. In primary cultured rabbit renal proximal tubule cells, activation of ERK1/2 reduced the expression of SGLT1 and, therefore, suppressed glucose uptake [36,37]. Vice versa, in mice with global SGLT1 knockout, myocardial ERK1/2 activity was increased under basal conditions and exhibited a significantly higher increment in response to early pressure overload than in wildtype littermates [3]. Therefore, an inverse relationship between ERK1/2 activity and SGLT1 expression might exist, which needs to be further elucidated.

Similar to ERK1/2, AMPK has also been proposed as a positive regulator of SGLT1 expression during acute myocardial ischemia-reperfusion injury [7], but its role in chronic HF-associated SGLT1 upregulation is unclear. Cardiomyocyte-specific overactivation of AMPK in mice, resulting in a distinct type of cardiomyopathy characterized by increased myocardial glycogen storage with severe LV dilation and dysfunction, was associated with increased expression of SGLT1 [8]. In these mice, pharmacological inhibition [8] or cardiomyocyte-specific knockdown [29] of SGLT1 rescued the cardiac phenotype by decreasing myocardial glucose uptake and glycogen accumulation. In the present study, AMPK activity was reduced in both HF models as shown by the reduction in the phosphorylation of ACC at the AMPK-specific Ser79 site. It is generally accepted that the function of AMPK is compromised in HF [38,39,40]. Yet, we found an increased expression of SGLT1. It is possible that AMPK contributes to SGLT1 upregulation in the heart during the early courses of hemodynamic overload, but as the activity of this kinase diminishes gradually in line with the development of chronic HF, other mechanisms might contribute to maintaining higher SGLT1 expression. Interestingly, neither cardiomyocyte-specific overexpression of SGLT1 nor its knockdown affected AMPK activity in mice [29].

Several previous studies causally linked the upregulation of SGLT1 to detrimental effects in the heart, including intracellular sodium ion overload [30], increased glucose uptake and subsequent glycogen accumulation [8,29], larger myocardial infarct size [7], and the development of myocardial pathological hypertrophy with fibrosis in response to chronic pressure overload [3]. Importantly, there is evidence that SGLT1 facilitates cellular nitro-oxidative stress and subsequent damage. In cell cultures of cardiomyocytes, overexpression of SGLT1 itself is sufficient to increase reactive oxygen species production via NADPH oxidase activation [7], whereas high-glucose-induced NADPH oxidase overactivation is dependent on SGLT1 [9]. Nox4 is the dominant NADPH oxidase isoform in the heart and TGF-β was shown to be a strong positive regulator of its expression [41]. In line with our present findings, previous studies showed that myocardial Nox4 is upregulated in both pressure [42] and volume overload-induced chronic HF [34]. In both cases, knockout of Nox4 resulted in lesser nitro-oxidative stress and protection against the development of adverse remodeling and LV dysfunction in response to hemodynamic overload [34,42]. In this study, we report for the first time that this increased expression of Nox4 in pressure and volume overload-induced HF is strongly correlated with the increase in expression of SGLT1, in line with substantially higher levels of nitro-oxidative stress and upregulation of TGF-β. Importantly, recent studies found that upregulation of SGLT1 is profibrotic [4,43], its knockdown in rats with T2DM reduces myocardial collagen expression and fibrotic accumulation [4]. Therefore, a functional link might exist between upregulation of myocardial SGLT1 in HF, increased nitro-oxidative stress, and the development of cardiac fibrosis, which needs to be further elucidated.

## 5. Conclusions

Taken together, myocardial SGLT1 protein expression is upregulated in both pressure and volume overload-induced chronic HF, indicating that it is a common pathology regardless of the type of hemodynamic overload. This upregulation of SGLT1 was observable despite maintained or reduced ERK1/2 and AMPK activity, suggesting that other mediators might contribute to inducing SGLT1 expression in chronic HF. Finally, there might be a link between upregulation of SGLT1 and increased myocardial nitro-oxidative stress, which warrants further investigation.

## Figures and Tables

**Table 1:** Morphometric analysis of sham-operated and hemodynamically-overloaded hearts.

	Sham-T	TAC	*p* Value	Sham-A	ACF	*p* Value
Body weight, BW (g)	520 ± 17	439 ± 17	0.002	642 ± 16	702 ± 22	0.042
Tibial length, TL (cm)	4.39 ± 0.04	4.21 ± 0.04	0.005	4.63 ± 0.03	4.67 ± 0.05	0.51
Heart weight, HW (g)	1.32 ± 0.05	2.72 ± 0.14	<0.001	1.56 ± 0.04	3.41 ± 0.18	<0.001
HW/TL (g/cm)	0.30 ± 0.01	0.65 ± 0.03	<0.001	0.34 ± 0.01	0.73 ± 0.04	<0.001
Lung weight, LW (g)	1.94 ± 0.07	4.06 ± 0.33	<0.001	2.06 ± 0.08	3.25 ± 0.18	<0.001
LW/TL (g/cm)	0.44 ± 0.01	0.97 ± 0.08	<0.001	0.44 ± 0.02	0.70 ± 0.04	<0.001

ACF = aortocaval fistula, TAC = transverse aortic constriction.

## Data Availability

Data is contained within the article.
